# Intestinal autophagy links psychosocial stress with gut microbiota to promote inflammatory bowel disease

**DOI:** 10.1038/s41419-019-1634-x

**Published:** 2019-09-30

**Authors:** Shu-Ling Wang, Bo-Zong Shao, Sheng-Bing Zhao, Xin Chang, Pei Wang, Chao-Yu Miao, Zhao-Shen Li, Yu Bai

**Affiliations:** 1 Department of Gastroenterology, Changhai Hospital, Second Military Medical University/Naval Medical University, Shanghai, China; 2 General Hospital of the Chinese People’s Liberation Army, Beijing, China; 3 Department of Pharmacology, Second Military Medical University/Naval Medical University, Shanghai, China

**Keywords:** Inflammation, Inflammatory bowel disease

## Abstract

Psychosocial stress is a critical inducing factor of inflammatory bowel diseases (IBD), while autophagy is a novel central issue of IBD development. The present study investigated the potential role of autophagy in stress-related IBD in patients and animal model. The correlation between psychosocial stress and intestinal autophagy was determined in 23 patients with IBD. Corticotropin-releasing hormone (CRH), a well-established inducer of psychosocial stress, was administrated in dextran sulfate sodium (DSS)-induced IBD mice and lipopolysaccharide (LPS)-stimulated bone marrow-derived macrophages (BMDM). In IBD patients, the autophagy markers beclin-1, LC3-II/I ratio, Atg16L1, and Atg4B were significantly enhanced. The psychosocial stress score was positively associated with the levels of beclin-1 and the LC3II/I ratio in intestinal biopsy specimens. In IBD mouse model, CRH significantly aggravated intestinal inflammation, increased Paneth cell metaplasia, and enhanced intestinal autophagy (beclin-1, Atg16L1, PIK3R4, and Atg4B upregulation; GAA, CTSD, and PPKAA1 downregulation). Additionally, the CRH-induced gut microbial dysbiosis was evidenced by a marked increase in the number of detrimental bacteria. In LPS-stimulated BMDM, CRH substantially increased M1/M2 polarization and thus promoted inflammation. In both IBD mice and LPS-treated BMDM, blockade of autophagy by chloroquine abrogated the unbeneficial effects of CRH, whereas autophagy inducer rapamycin resulted in a pronounced protective effect against IBD lesion. Our data demonstrate that psychosocial stress may link the enhanced intestinal autophagy by modulating gut microbiota and inflammation to aggravate IBD. These data indicate autophagy as a promising therapeutic target for psychosocial stress-related IBD.

## Introduction

Inflammatory bowel disease (IBD) is characterized with chronic and recurrent inflammation in the intestinal mucosa, including ulcerative colitis (UC) and Crohn’s disease (CD)^1,2^. Psychosocial stress has been reported to contribute to the pathogenesis and progression of IBD via damage to the intestinal defense system^3,4^. In the central nervous system, the physiological response to stress lies in the triggering of the hypothalamic-pituitary-adrenal axis, thus releasing corticotropin-releasing hormone (CRH) for the regulation of neuroendocrine function and internal organs immune reaction^5,6^. CRH is also located in the peripheral tissues, including the digestive system organs and cells such as Paneth cells, macrophages, and mast cells, directly regulating the intestinal inflammatory and digestive tract inflammation. Peripheral CRH administration reproduced the effect of psychosocial stress and triggered for onset and development of IBD^7^. Additionally, inhibition of CRH receptor 1 protected against colonic injury and promoted epithelium repair^8^. Thus, the CRH signaling plays a crucial role in the so-called “brain–gut interactions” in IBD^9^. However, the molecular mechanisms of how CRH induces IBD have not been fully elucidated.

It is commonly acknowledged that inflammation is the most important event in the pathophysiology of IBD. Importantly, it has been well-accepted that psychosocial stress links to intestinal inflammation because the nervous system can affect immune function at both the systemic and gut mucosal levels^10^. During the intestinal barrier disruption and inflammation development, it becomes gradually apparent that autophagy and gut microbiota are two new crucial players. Autophagy is a vital and fundamental cellular process. The biological function of autophagy is to degrade the long-lived and misfolded proteins, as well as useless cellular organelles into basal nutrient elements such as amino acid for recycling^11,12^. In the digestive system, baseline autophagy is crucial for the maintenance of intestinal homeostasis. The autophagy process within the epithelium controls inflammation-induced apoptosis and protects barrier integrity to limit chronic intestinal inflammation^13^. Deletion of the autophagy gene *Atg1611* in the T cells in mice resulted in an intensive spontaneous intestinal inflammation^14^. In contrast, it was also reported that abnormal induction of intestinal autophagy contributed to the aggravation of IBD^15,16^. As a result, the role of autophagy in IBD is controversial and requires further study.

Gut microbiota is another critical factor involved in IBD onset and development. The gastrointestinal tract is the primary site of interaction between the host immune system and microorganisms. The microorganisms known collectively as the “gut microbiota” colonize the gastrointestinal tract and influence IBD^17^. Gut bacterial dysbiosis induce the development of chronic intestinal inflammation and subsequent Paneth cell malfunction^18^. Analysis of microbiota of intestinal biopsies and stool samples from 231 IBD and healthy subjects demonstrate that there is a major shift in the oxidative stress pathways, as well as decreased carbohydrate metabolism and amino acid biosynthesis in favor of nutrient transport and uptake in gut microbiota with IBD^19^. In a rat stress model, gut microbiota was altered at 3 h after CRH administration^20^.

The present study posits that autophagy may be involved in the psychosocial stress-related IBD. Our results demonstrate that the over-induced autophagy in intestines from IBD patients is associated with high psychosocial stress. Moreover, CRH-mediated stress enhances autophagy, changes gut microbiota composition, promotes inflammation, and exacerbates IBD severity in the IBD mice model. These data highlight the role of autophagy in the pathological mechanisms of IBD.

## Results

### IBD patients with high psychosocial stress have enhanced intestinal autophagy

**Fig. 1:**
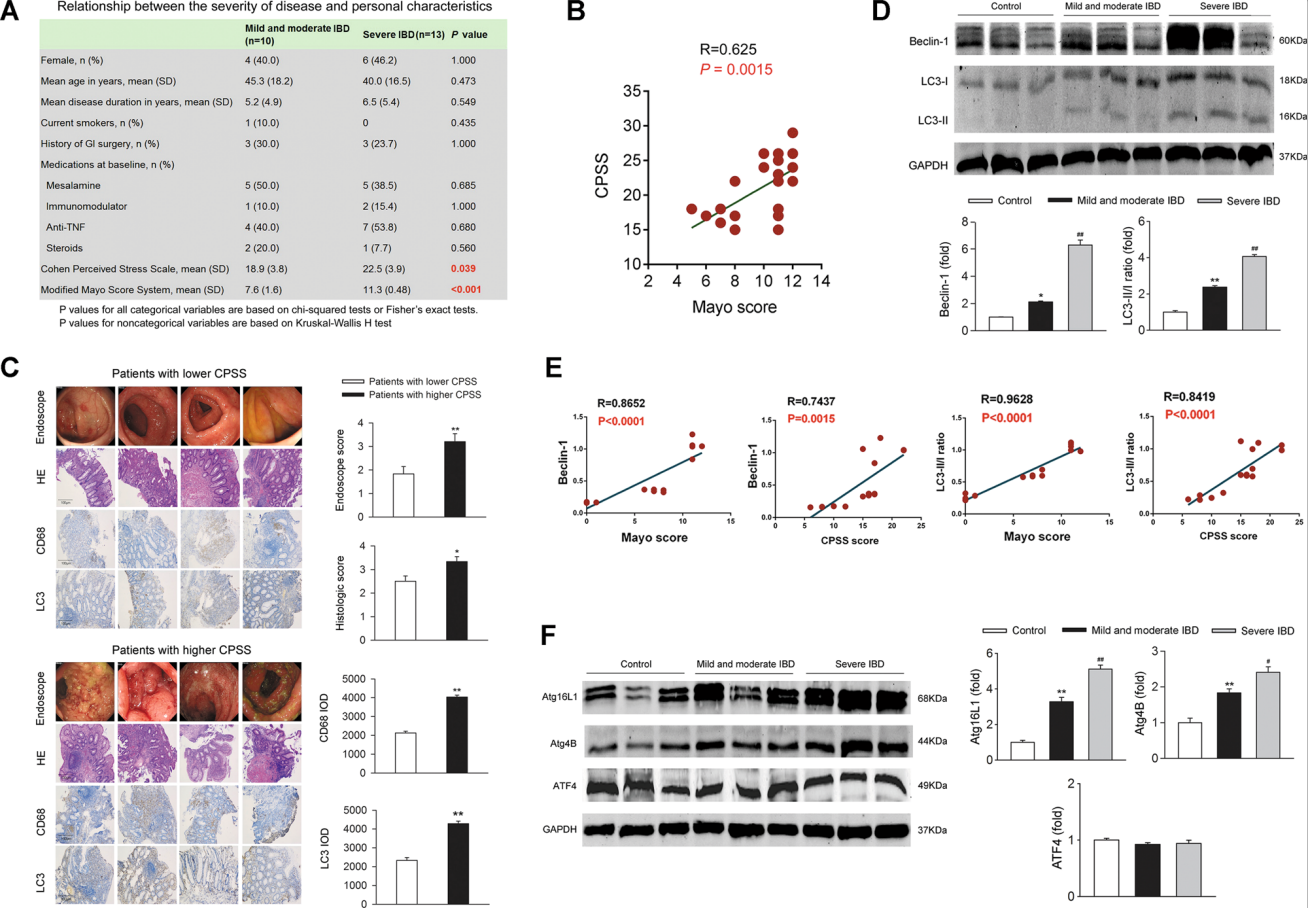
Stress contributes to the aggravation of IBD and enhancement of autophagy in IBD patients. Ten mild/moderate IBD and 13 severe IBD patients were enrolled. The basic characteristics, and endoscopic pictures were obtained during the colonoscopy. The colonic biopsy tissue was fixed, and H&E staining was used for assessment of histological changes. Immunohistochemical staining for LC3 was used for identification of autophagy and immunohistochemical staining for CD68 for detection of M1. Western blotting was applied for the detection of autophagy-related Beclin-1 and LC3-II/I ratio and correlation between autophagy-related proteins (Beclin-1 and LC3-II/I ratio) and IBD severity index (IBD activity and score) was analyzed by correlation analysis. **a**, **b** Compared to the mild and moderate IBD group, the severe IBD patient group showed a significant increase in CPSS (*P* = 0.039) and there was a moderate relationship between the CPSS and Mayo Score (*R* = 0.625, *P* = 0.0015). **c** Compared to the patients with lower CPSS, those with higher CPSS showed aggravated inflammatory infiltration in the colonoscopic view and HE staining, as well as enhancement of the levels of CD68 and LC3 in the colonic tissue (*n* = 6 per group). ^*^P < 0.05 vs. the patients with low CPSS, ^**^P < 0.01 vs. the patients with low CPSS. **d** Western blotting analysis for Beclin-1, LC3-II/I ratio in biopsy specimens from the healthy controls, mild/moderate IBD patients, and severe IBD patients. ^*^P < 0.05, ^**^P < 0.01 vs. the control group; ^#^*P* < 0.05, ^##^*P* < 0.01 vs. the mild and moderate IBD group. **e** Correlation analysis between psychosocial stress and autophagy (evaluated with Beclin-1 and LC3-II/I ratio) and correlation analysis between IBD score and autophagy (evaluated with Beclin-1 and LC3-II/I ratio). ^*^P < 0.05, ^**^P < 0.01 vs. the control group; ^#^*P* < 0.05, ^##^*P* < 0.01 vs. the mild and moderate IBD group. **f** Western blotting analysis for Atg16L1, Atg4B, and ATF4 in biopsy specimens from health control, mild/moderate IBD patients and severe IBD patients (*n* = 6 per group). ^*^P < 0.05, ^**^P < 0.01 vs. the control group; ^#^*P* < 0.05, ^##^*P* < 0.01 vs. the mild and moderate IBD group

The potential relationship between autophagy and psychosocial stress was evaluated in IBD patients. Ten patients with mild/moderate IBD and 13 patients with severe IBD were recruited, and their basic characteristics, endoscopic images, and colonic tissue during the colonoscopy procedures were collected. The mean modified Mayo Score system in mild/moderate IBD and severe IBD were 7.6 and 11.3, respectively (*P* < 0.001). Comparison of the basic characteristics was performed between the patients in the two groups. No significant differences were found in the baseline characteristics, including sex, age, disease duration, smoking history, and medications (Fig. 1a). However, the Cohen Perceived Stress Scale (CPSS) of the severe IBD patients was significantly higher than that of the mild/moderate IBD patients. A Spearman rank correlation analysis between CPSS and the Mayo score showed a positive relationship between the perceived stress and the severity of IBD in the patients (Fig. 1b).

To further explore the relationship between autophagy and IBD severity in IBD patients, we chose six IBD patients with the lowest CPSS and six IBD patients with the highest CPSS from the all 23 individuals. The endoscopic images, histology (H&E staining), monocytes/macrophages infiltration (CD68 immunohistochemistry), and autophagy (LC3 immunohistochemistry) were shown in Fig. 1c. Colonoscopy images and histologic detection confirmed the increase in inflammatory infiltration in the colon of patients with a higher CPSS compared to the patients with lower level of perceived stress. Intriguingly, the biopsy specimens from the patients with higher CPSS showed a marked increase in LC3 staining, which is a molecular marker of autophagy.

In addition, the protein levels of autophagy-related Beclin-1 and LC3-II/I ratio in intestinal biopsy specimens from patients with mild/moderate IBD were higher than those in health control individuals. In the intestinal biopsy specimens from severe IBD patients, the levels of Beclin-1 and LC3-II/I ratio were much higher (Fig. 1d). Positive correlations between the IBD score and the Beclin-1 or LC3-II/I ratio were found. Moreover, the psychosocial stress score was also positively correlated with the levels of Beclin-1 or LC3-II/I ratio (Fig. 1e). In addition, the protein levels of autophagy-related Atg16L1, Atg4B, and ATF4 in intestinal biopsy specimen from control, mild/moderate IBD patients and severe patients were analyzed. The protein expression of Atg16L1 and Atg4B increased in IBD patients. Moreover, they displayed much higher expression in severe IBD patients compared with mild/moderate IBD patients (Fig. 1f). ATF4 was not altered among these groups. These results indicated that the IBD patients with high psychosocial stress had enhanced intestinal autophagy.

### Administration of CRH deteriorates the severity of IBD

**Fig. 2:**
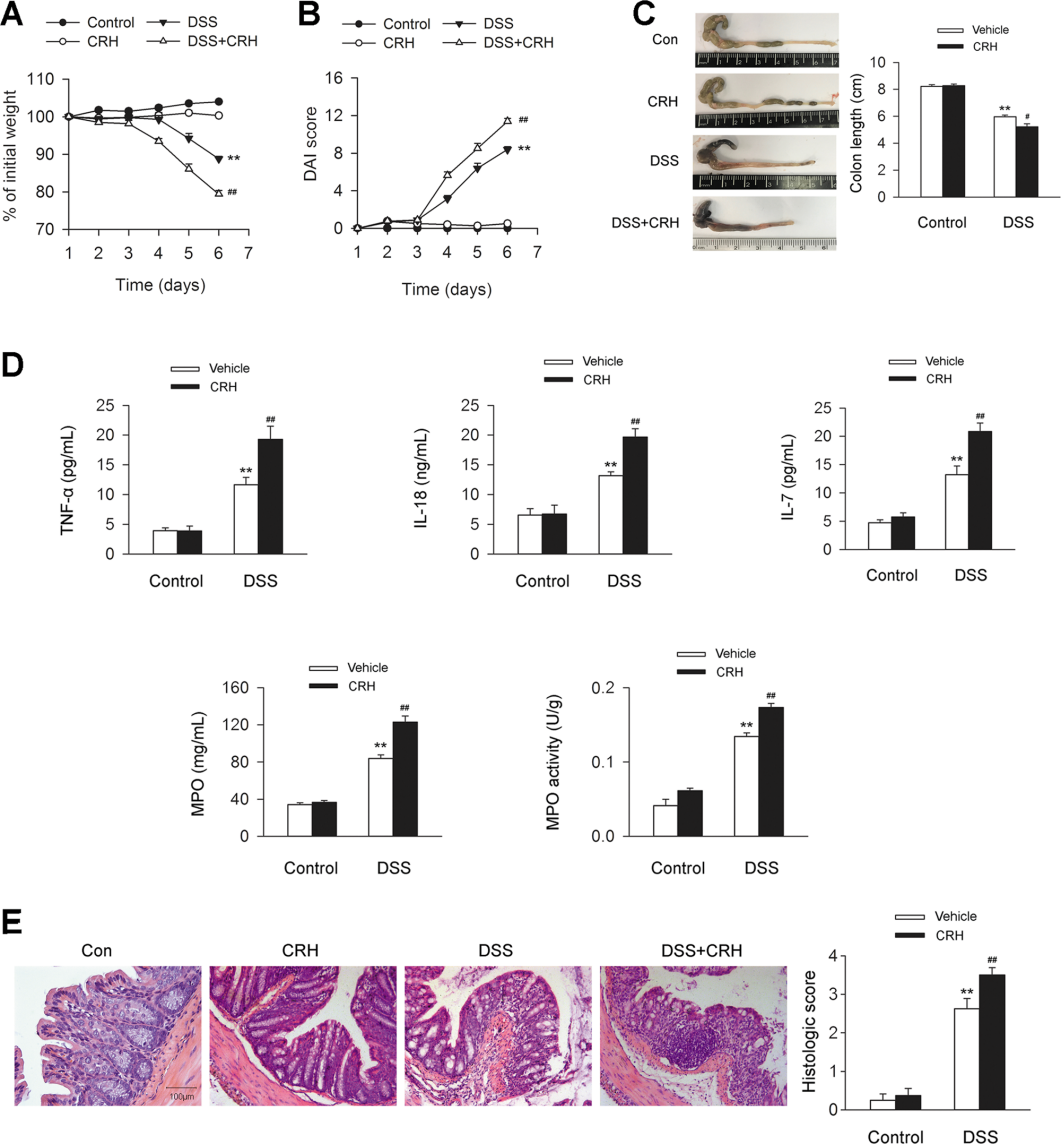
Severity of IBD is aggravated by peripheral administration of CRH. DSS (3%) was given to C57BL/6 mice for 6 days while water was given to the control group. For certain groups, CRH was intraperitoneally given at the dose of 50 μg/kg body weight from day 1 to day 6 and saline was injected as vehicle. Body weight, the presence of occult or gross blood per rectum, stool consistency, and colon length were determined by two investigators blinded to the treatment groups. **a**–**c** Compared to the DSS + Vehicle group, the mice in the DSS + CRH group exhibited significant deterioration in body weight loss, bloody stool score, and colon length (*n* = 8 per group). ^**^P < 0.01 vs. the control group, ^#^*P* < 0.05 vs. the DSS + Vehicle group, ^##^*P* < 0.01 vs. the DSS + Vehicle group. **d** ELISA and the O-dianisidine method were used for the detection of the levels of TNF-α, IL-18, IL-7, MPO, and MPO activity in the left colon. Compared to the DSS + Vehicle group, the mice in the DSS + CRH group showed aggravated enhancement of the levels of TNF-α, IL-18, IL-7, MPO, and MPO activity in serum (*n* = 6 per group). ^**^P < 0.01 vs. the control group, ^##^*P* < 0.01 vs. the DSS + Vehicle group. **e** The left edge of the left colon was isolated and fixed and H&E staining was used for the detection of histological score. Compared to the DSS + Vehicle group, the mice in the DSS + CRH group showed aggravated inflammatory infiltration in the left colon (*n* = 8 per group). ^**^P < 0.01 vs. the control group, ^##^*P* < 0.01 vs. the DSS + Vehicle group

To further investigate the effect of psychosocial stress on autophagy during IBD, CRH was injected peripherally in IBD mice to reproduce the effect of psychosocial stress. Mice receiving 3% DSS for 7 days developed a severe illness characterized by the presence of sustained weight loss (Fig. 2a) and bloody diarrhea (Fig. 2b), and shortened colon length (Fig. 2c). Notably, the administration of CRH in DSS-treated mice further aggravated these IBD symptoms (Fig. 2a–c). The levels of proinflammatory factors, including TNF-α, IL-18, and IL-7, were elevated in IBD mice, which were further enhanced by CRH. MPO, a marker for tissue neutrophil content, as well as its activity, were also further enhanced by CRH (Fig. 2d). The histologic morphology analysis of inflammatory infiltration of the left colon confirmed the increased inflammatory reaction in tissue (Fig. 2e). These results demonstrated that the administration of psychosocial stress mimic agent CRH aggravated the progress of IBD.

### Administration of CRH enhances Paneth cell metaplasia in the epithelium of the left colon from IBD mice

**Fig. 3:**
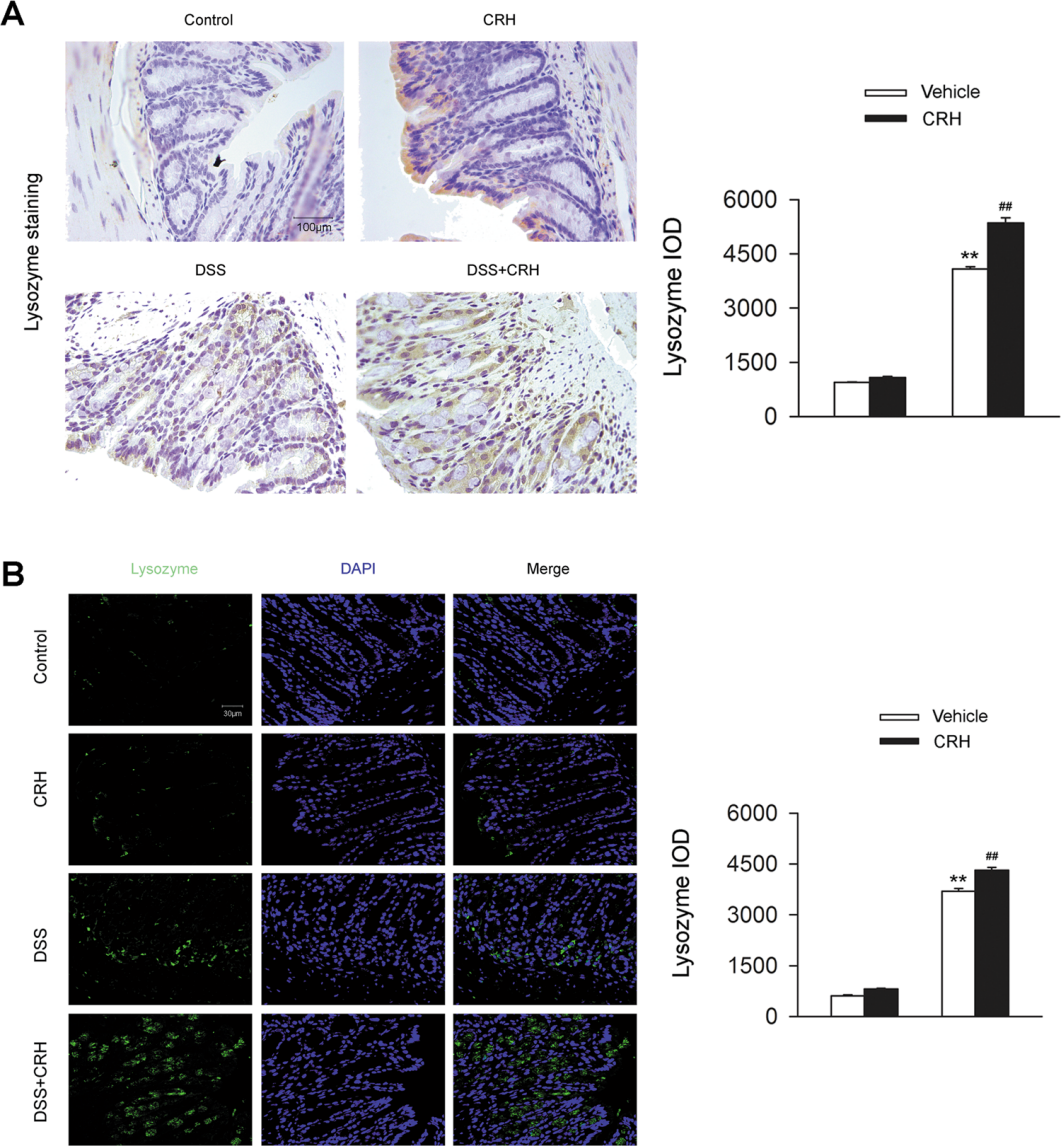
Paneth cell metaplasia is enhanced by peripheral administration of CRH in the epithelium of left the colon from IBD mice. DSS (3%) was given to C57BL/6 mice for 6 days while water was given to the control group. For certain groups, CRH was intraperitoneally given at the dose of 50 μg/kg body weight from day 1 to day 6 and saline was injected as vehicle. **a** The left edge of the left colon was separated and fixed and immunohistochemical staining was used for the detection of Paneth cell. IOD values were analyzed. Compared to the DSS + Vehicle group, the mice in the DSS + CRH group showed enhancement of Paneth cell metaplasia in the epithelium of the left colon (*n* = 6 per group). ^**^P < 0.01 vs. the control group, ^##^*P* < 0.01 vs. the DSS + Vehicle group. **b** The left edge of the left colon was further detected by immunofluorescence staining and IOD values were analyzed. Compared to the DSS + Vehicle group, the mice in the DSS + CRH group showed enhancement of Paneth cell metaplasia in the epithelium of the left colon (*n* = 6 per group). ^**^P < 0.01 vs. the control group, ^##^*P* < 0.01 vs. the DSS + Vehicle group

Paneth cells are normally distributed in the epithelium of the small intestine. However, on the occurrence of IBD, Paneth cell metaplasia have been observed in the epithelium of colonic tissue^21^. Thus, we detected the metaplasia of Paneth cells in the epithelium during the occurrence of IBD. Mice receiving 3% DSS for 7 days showed an obvious metaplasia of the Paneth cells in the epithelium of IBD mice models in the left colon (Fig. 3a, b). Administration of CRH further increased Paneth cell metaplasia (Fig. 3a, b). These data indicated the peripheral CRH administration promoted Paneth cell metaplasia in the left colon of IBD.

### Administration of CRH disturbs the intestinal microbiota balance in IBD mice

**Fig. 4:**
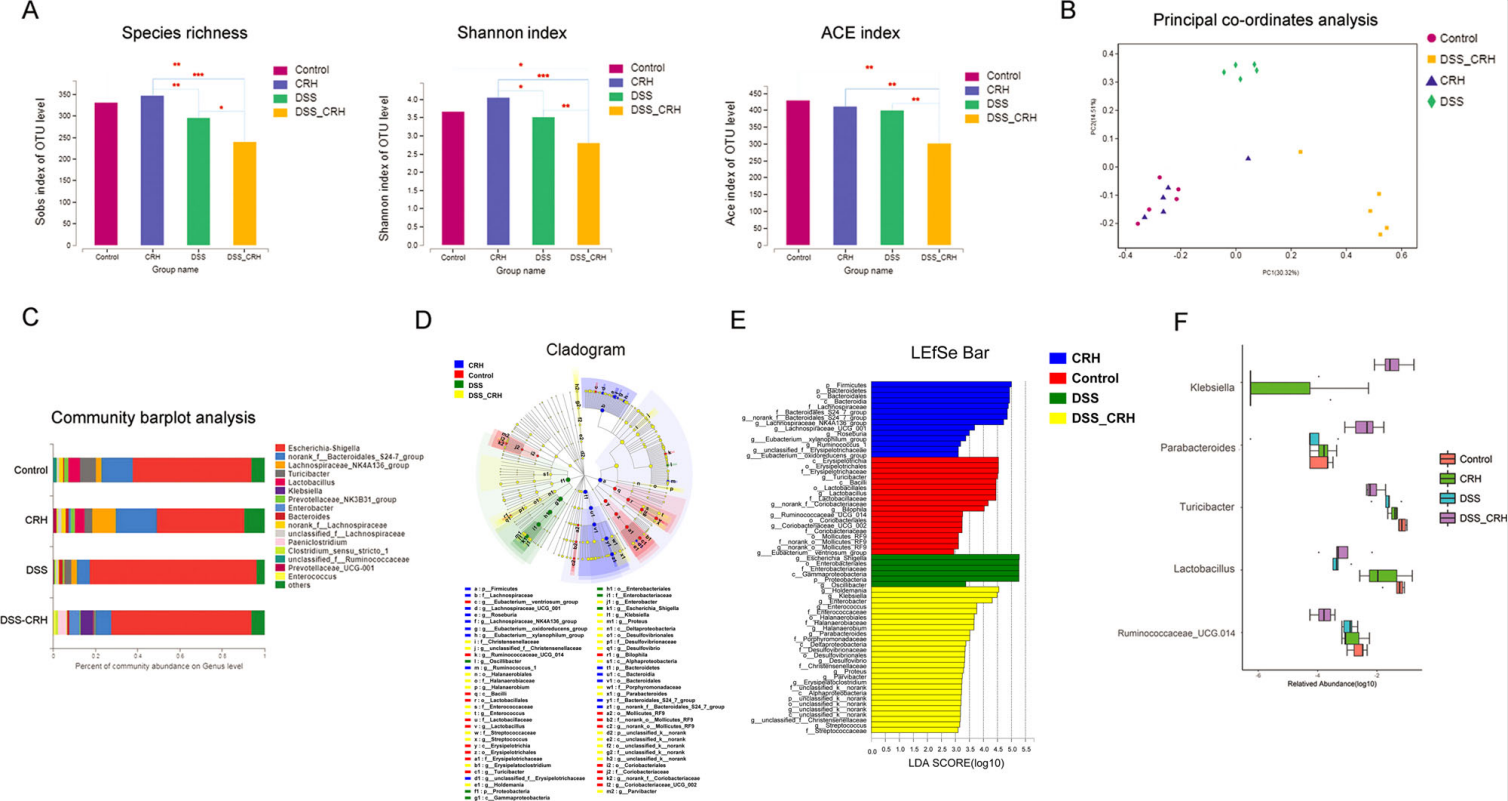
Intestinal microflora homeostasis is deteriorated by peripheral administration of CRH in IBD mice. DSS (3%) was given to C57BL/6 mice for 6 days while water was given to the control group. For certain groups, CRH was intraperitoneally given at the dose of 50 μg/kg body weight from day 1 to day 6 and saline was injected as vehicle. Fecal samples were analyzed by performing high-throughput 16S rRNA gene sequencing (*n* = 5). **a** α-Diversity, illustrated by microbiota richness (number of observed operational taxonomic unit (OTU)), Shannon and ace index were reduced in the DSS group, and peripheral administration of CRH aggravated this downward trending (*P* = 0.035, 0.012, and 0.094, respectively, Wilcoxon rank-sum test analysis), ^*^P < 0.05, ^**^P < 0.01. **b** Principal coordinate analysis (PCoA) of the overall microbial composition of the DSS and DSS + CRH groups deviated from the Control and CRH groups (ANOSIM R: 0.7887, *P* = 0.001). **c** Compared to the control group, microbiome alterations at the genus level in the DSS group were significantly changed, and this trend was further obvious in the DSS + CRH group. **d**, **e** Linear discriminant analysis (LDA) effect size (LEfSe) analysis illustrated marked bacterial differences in fecal microbiota among these four groups. **f** The abundance of *Turicibacter, Ruminococcaceae, and Lactobacillus* were decreased, while the genera Klebsiella and Parabacteroides were increased in the DSS group. Moreover, peripheral administration of CRH further aggravated these changes in IBD

We then detected the influence of peripheral CRH in the homeostasis of intestinal microflora in IBD mice by performing high-throughput 16S rRNA gene sequencing. Metrics was used to assess differences in microbial alpha diversity (species richness, Shannon, and ace index). Mice receiving 3% DSS for 6 days had lower microbial alpha diversity than the control group, and the administration of CRH further aggravated this downward trend (Fig. 4a). Moreover, statistical difference was detected between the DSS + CRH and DSS samples. A principal co-ordinates analysis revealed that the overall microbial composition of the DSS and DSS + CRH groups deviated from the Control and CRH groups (ANOSIM R: 0.7887, *P* = 0.001, Fig. 4b). A prominent feature of the fecal microbiome was that the composition in the DSS group significantly differed from the control group (Fig. 4c). The *norank_f_Bacteroidales_S24-7_*group, *Lachnospiraceae_NIK4A136_*group, *Turicibacter*, and *Lactobacillus* in DSS mice significantly decreased while the *Escherichia-Shigella* and *Klebsiella* were much more abundant. Moreover, administration of CRH promoted this imbalance. A supervised comparison of the microbiota between the DSS + CRH and DSS groups was performed by utilizing the cladogram analysis (Fig. 4d) and linear discriminant analysis (LDA) effect size (LEfSe) analysis (Fig. 4e). Marked differences were found (Fig. 4d, e). The current study particularly focused on the differences in the taxa at the genus level. A significantly higher proportions of the *genus Turicibacter, Ruminococcaceae, and Lactobacillus* were detected in the control group than those in DSS group, which was similar to previous research^22–24^, while a great abundance of the genera *Klebsiella*, *Parabacteroides* was observed in the DSS group, which was also validated by other studies^25,26^. Moreover, the administration of CRH further aggravated these changes in IBD (Fig. 4f).

### Peripheral administration of CRH further enhances intestinal autophagy in IBD

**Fig. 5:**
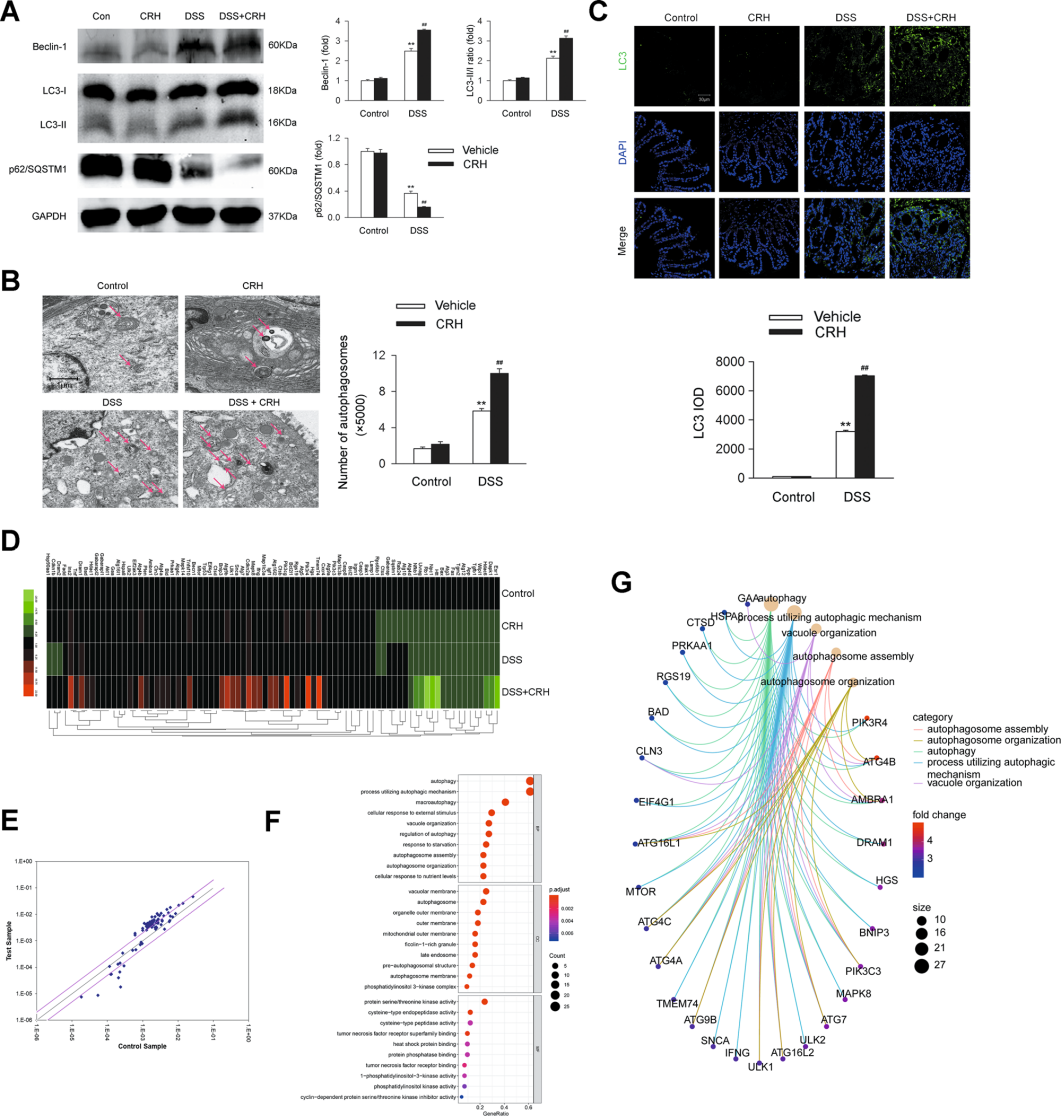
The level of autophagy is increased by peripheral administration of CRH in the left colon from IBD mice. DSS (3%) was given to C57BL/6 mice for 6 days while water was given to the control group. For certain groups, CRH was intraperitoneally given at the dose of 50 μg/kg body weight from day 1 to day 6 and saline was injected as vehicle. **a** The left colon was separated and the levels of Beclin-1, LC3-II/I ratio, and p62/SQSTM1 were analyzed by western blotting. Compared to the DSS + Vehicle group, the mice in the DSS + CRH group showed an increase in Beclin-1 and LC3-II/I ratio while a decrease in p62/SQSTM1 (*n* = 5 per group). ^**^P < 0.01 vs. the control group, ^##^*P* < 0.01 vs. the DSS + Vehicle group. Electronic microscope was used for the detection of autophagosome number (**b**) and immunofluorescence staining for LC3 was used for the analysis of autophagy level (**c**). Compared to the DSS + Vehicle group, the mice in the DSS + CRH group showed an increase in the number of autophagosome and LC3 dots (*n* = 6 per group). ^**^P < 0.01 vs. the control group, ^##^*P* < 0.01 vs. the DSS + Vehicle group. The left colon was separated and the total RNA (125 ng) was extracted and reverse transcribed to cDNA using miScript II RT kit (Qiagen, Valencia, CA, USA), and the expression profiles of autophagy gene, including a total of 84 genes were analyzed. **d** Heatmap displaying the relative expression of autophagy-related genes in the control, CRH, DSS + Vehicle and DSS + CRH groups (*n* = 3 per group). **e** Relative gene expression profiles between the DSS + Vehicle vs. the DSS + CRH mice (*n* = 3). The red lines indicate positive or negative fold changes (upregulated twofold or downregulated 0.5-fold) for selected genes in the DSS vs. DSS + CRH mice. **f** Most significant increased genes were mainly associated with autophagy, the process utilizing autophagic mechanism, and macroautophagy. **g** Compared to the DSS group, the levels of several proteins vital in the induction and regulation of autophagy were significantly changed (red nodes for genes with higher expression and blue nodes for genes with lower expression). Compared to the DSS + Vehicle group, several proteins positively correlated to autophagy such as PIK3R4, Atg4B, and DRAM1 were upregulated and those negatively correlated to autophagy such as GAA, HSP, CTSD, PPKAA1 were downregulated in the DSS + CRH group

It was previously demonstrated that the autophagy process played an important role in the pathogenesis and progression of IBD^27,28^. The present study investigated whether administration of CRH affected the level of colonic autophagy. The mice receiving 3% DSS for 6 days showed an increase in the level of Beclin-1 and LC3-II/I ratio, as well as a decrease in p62/SQSTM1. Administration of CRH led to a further increase in colonic autophagy (Fig. 5a). Similar trends in changes such as those of Beclin-1 and LC3-II/I ratio were detected in the number of autophagosomes (Fig. 5b) and LC3 dots (Fig. 5c) in the left colon. We also used an autophagy-specific microarray to monitor the expression profiles of autophagy gene, including a total of 84 genes (Fig. 5d–g). The results showed that compared to the DSS group, the levels of several vital autophagy-related genes were significantly increased in the DSS + CRH group (Fig. 5d–f). Compared to the DSS group, the levels of several proteins vital in the induction and regulation of autophagy were significantly changed (Fig. 5g). We then ran western blotting analysis for the detection of the levels of proteins with the most significant changes. We found that compared to the DSS + Vehicle group, the DSS + CRH group increased the levels of Beclin-1, Atg16L1, PIK3R4, and Atg4B but decreased the levels of GAA, CTSD, and PPKAA1, while the level of ATF4 was not changed (Supplementary Fig. 1). Taken together, these results indicated that peripheral CRH further enhanced intestinal autophagy in IBD.

### Blockade of autophagy by chloroquine attenuates CRH-induced colonic damage and Paneth cell metaplasia in IBD mice

**Fig. 6:**
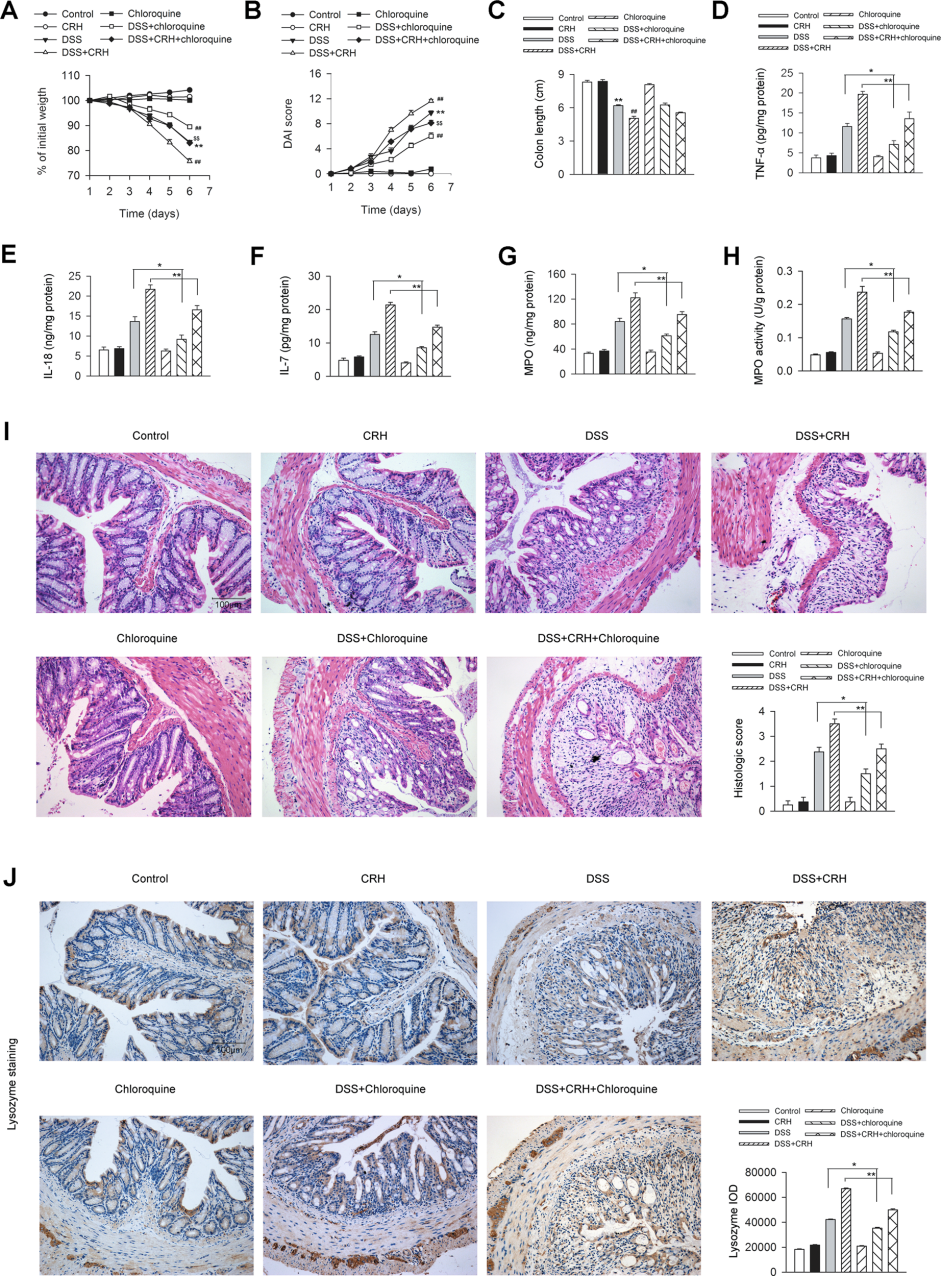
Chloroquine ameliorates CRH-induced colonic damage and Paneth cell metaplasia in IBD mice. DSS (3%) was given to C57BL/6 mice for 6 days while water was given to the control group. For certain groups, CRH (50 μg/kg body weight) and/or chloroquine (60 mg/kg body weight) was intraperitoneally given from day 1 to day 6 and saline was injected as vehicle. **a**–**c** Body weight, the presence of occult or gross blood per rectum, stool consistency, and colon length were determined by two investigators blinded to the treatment groups. Compared to the DSS + Vehicle group, the mice in the DSS + CRH group exhibited significant deterioration in body weight loss, bloody stool score, and colon length, while the administration of chloroquine largely attenuated the aggravated effect of CRH (*n* = 8 per group). ^**^P < 0.01 vs. the control group, ^##^*P* < 0.01 vs. the DSS + Vehicle group, ^$$^*P* < 0.01 vs. the DSS + CRH group. **d**–**h** The left edge of the left colon was isolated and the levels of TNF-α, IL-18, IL-7, MPO, and the MPO activity in the left colon were analyzed by ELISA and O-dianisidine method. Compared to the DSS + Vehicle group, the mice in the DSS + CRH group showed aggravated colonic inflammation, while the administration of chloroquine largely attenuated the effect of CRH (*n* = 6 per group). ^*^P < 0.05, ^**^P < 0.01. **i** The left edge of the left colon was isolated and fixed and H&E staining was used for the detection of histological score. Compared to the DSS + Vehicle group, the mice in the DSS + CRH group showed aggravated inflammatory infiltration in the left colon, while the administration of chloroquine largely attenuated the effect of CRH (*n* = 8 per group). ^*^P < 0.05, ^**^P < 0.01. **j** The left edge of the colon was separated and fixed and immunohistochemical staining was used for the detection of Paneth cell. IOD values were analyzed. Compared to the DSS + Vehicle group, the mice in the DSS + CRH group showed enhancement of Paneth cell metaplasia in the epithelium of the left colon, while the administration of chloroquine largely attenuated the effect of CRH (*n* = 6 per group). ^*^P < 0.05, ^**^P < 0.01

To determine whether the autophagy process was involved in the CRH-induced intestinal damage in IBD mice, we used an autophagy inhibitor, chloroquine, for the pharmacological blockade of the autophagy process and detected the severity of IBD. The administration of CRH significantly aggravated the severity of IBD in body weight loss (Fig. 6a), bloody stool score (Fig. 6b), and colon length (Fig. 6c). These effects were obviously blocked by chloroquine (Fig. 6a–c). Additionally, chloroquine (60 mg/kg) significantly reversed the enhanced TNF-α (Fig. 6d), IL-18 (Fig. 6e), and IL-7 expression (Fig. 6f). Accordingly, chloroquine suppressed the expression and activity of inflammatory infiltration marker MPO (Fig. 6g, h) and inflammatory infiltration (Fig. 6i). In addition, the administration of CRH significantly enhanced Paneth cell metaplasia in the epithelium, while chloroquine largely attenuated the effect (Fig. 6j). We further demonstrated that chloroquine attenuated CRH-induced enhancement of autophagy in the left colon from IBD mice (Supplemental Fig. 1). All of these data indicated that autophagy was required for the CRH-induced colonic damage and Paneth cell metaplasia in IBD mice.

### Induction of autophagy by rapamycin aggravated CRH-induced Paneth cell metaplasia but not colonic damage in IBD mice

**Fig. 7:**
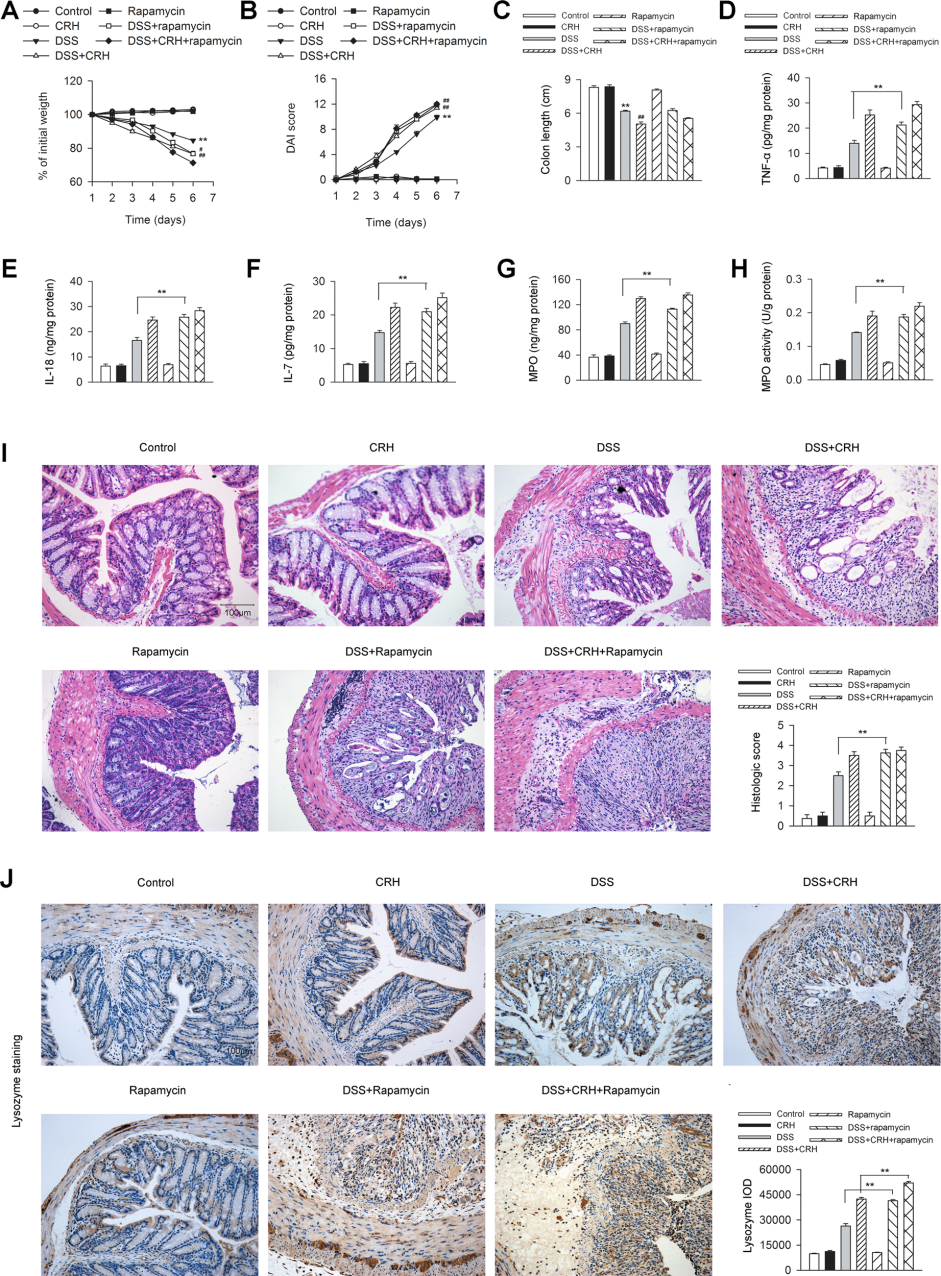
Rapamycin aggravated CRH-induced Paneth cell metaplasia but not colonic damage in IBD mice. DSS (3%) was given to C57BL/6 mice for 6 days while water was given to the control group. For certain groups, CRH (50 μg/kg body weight) and/or rapamycin (1.25 mg/kg body weight) was intraperitoneally given from day 1 to day 6 and saline was injected as vehicle. **a**–**c** Body weight, the presence of occult or gross blood per rectum, stool consistency, and colon length were determined by two investigators blinded to the treatment groups. Compared to the DSS + Vehicle group, the mice in the DSS + CRH group exhibited significant deterioration in body weight loss, bloody stool score, and colon length, which were not significantly affected by rapamycin (*n* = 8 per group). ^*^P < 0.05 vs. the control group. ^**^P < 0.01 vs. the control group. ^##^*P* < 0.01 vs. the DSS + Vehicle group. **d**–**h** The left edge of the left colon was isolated and the levels of TNF-α, IL-18, IL-7, MPO, and MPO activity in the left colon were analyzed by ELISA and the O-dianisidine method. Compared to the DSS + Vehicle group, the mice in the DSS + CRH group showed aggravated colonic inflammation, which were not significantly affected by rapamycin (*n* = 6 per group). ^**^P < 0.01. **i** The left edge of the colon was isolated and fixed and H&E staining was used for the detection of histological score. Compared to the DSS + Vehicle group, the mice in the DSS + CRH group showed aggravated inflammatory infiltration in the left colon, which were not significantly affected by rapamycin (*n* = 8 per group). ^**^P < 0.01. **j** The left edge of the left colon was separated and fixed and immunohistochemical staining was used for the detection of Paneth cell. IOD values were analyzed. Compared to the DSS + Vehicle group, the mice in the DSS + CRH group showed enhancement of Paneth cell metaplasia in the epithelium of the left colon, while the administration of rapamycin further aggravated the effect of CRH (*n* = 6 per group). ^**^P < 0.01

We further investigated the involvement of autophagy in the CRH-induced intestinal damage and Paneth cell metaplasia through the induction of autophagy by rapamycin. Inducing autophagy by rapamycin did not significantly aggravate the detrimental effects led to by administration of CRH in body weight loss (Fig. 7a), bloody stool score (Fig. 7b), colon length (Fig. 7c), and inflammatory infiltration (Fig. 7i) in the left colon, as well as inflammatory reaction (Fig. 7d–h). However, rapamycin significantly aggravated the effect of CRH-mediated enhancement of Paneth cell metaplasia in the epithelium (Fig. 7j). Taken together, these data indicated that autophagy was more closely involved in the involvement of CRH-induced Paneth cell metaplasia compared to intestinal damage in IBD mice.

### Blockade of autophagy by chloroquine attenuates CRH-induced enhancement of the M1/M2 ratio in the left colon from IBD mice and in murine bone marrow-derived macrophage (BMDM) under the challenge of lipopolysaccharide (LPS)

**Fig. 8:**
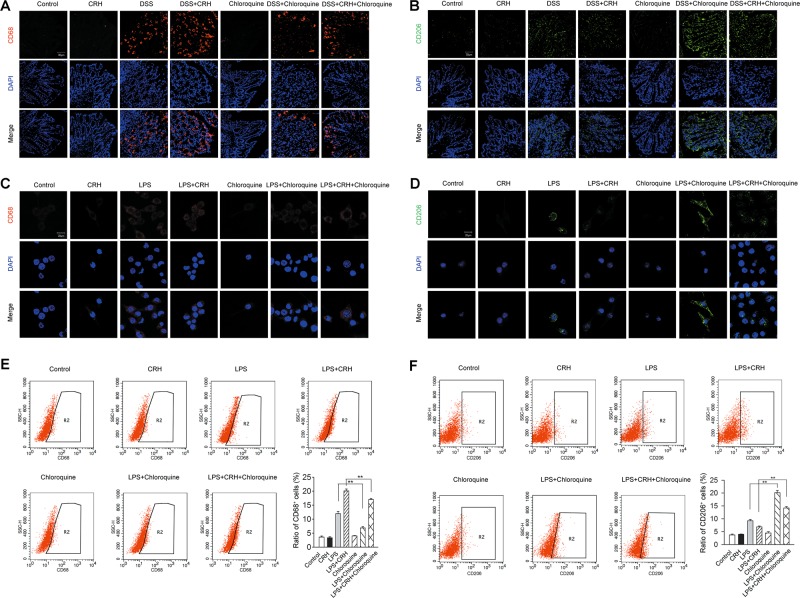
Chloroquine attenuates CRH-induced enhancement of M1/M2 ratio in the left colon from IBD mice in murine BMDM under the challenge of LPS. **a**, **b** DSS (3%) was given to C57BL/6 mice for 6 days while water was given to the control group. For certain groups, CRH (50 μg/kg body weight) and/or chloroquine (60 mg/kg body weight) was intraperitoneally given from day 1 to day 6 and saline was injected as vehicle. The left edge of colon was separated, and fixed and immunofluorescence staining was used for the detection of CD68 (M1 cells) and CD206 (M2 cells). Compared to the DSS + Vehicle group, the mice in the DSS + CRH group had an increased ratio of CD68^+^ cells and a decreased ratio of CD206^+^ cells in the left colon, while the administration of chloroquine largely attenuated the effect of CRH (*n* = 6 per group). **c**, **d** Murine BMDM were obtained and stimulated with LPS (100 ng/ml), CRH (10^−8^ M), and/or chloroquine (10 μM), and immunofluorescence staining was used for the detection of CD68 (M1 cells), and CD206 (M2 cells). Compared to the LPS + Vehicle group, the LPS + CRH group had an increased ratio of CD68^+^ cells and a decreased ratio of CD206^+^ cells, while the administration of chloroquine largely attenuated the effect of CRH (*n* = 6 per group). **e**, **f** Murine BMDM were obtained and stimulated with LPS (100 ng/ml), CRH (10^−8^ M), and/or chloroquine (10 μM). FACS analysis was conducted for the detection of CD68^+^ cell and CD206^+^ cell ratios. Compared to the LPS + Vehicle group, the LPS + CRH group had an increased ratio of CD68^+^ cells and a decreased ratio of CD206^+^ cells, while the administration of chloroquine largely attenuated the effect of CRH (*n* = 6 per group). ^**^P < 0.01.

We then detected whether the change in the M1/M2 ratio, a vital factor of the induction of inflammatory reaction, was involved in this process. We used CD68 and CD206 for the labeling of M1 and M2 cells, respectively. The administration of CRH significantly increased the ratio of CD68^+^ cells while decreased that of CD206^+^ cells in the left colon from IBD mice. However, those effects were attenuated through the blockade of the autophagy process by chloroquine (Fig. 8a, b). Similar trends in changes were observed in murine BMDM under the challenge of LPS in the ratios of CD68^+^ and CD206^+^ cells (Fig. 8c–f). In addition, chloroquine attenuated CRH-induced increase in CD68 messenger RNA (mRNA) and decrease in CD206 mRNA in LPS-treated BMDM (Supplemental Fig. 2). Taken together, these data indicated that autophagy was involved in CRH-induced enhancement of the M1/M2 ratio in the left colon from IBD mice and in murine BMDM under the challenge of LPS.

## Discussion

Various studies have illustrated that patients with IBD symptoms are more likely to have higher levels of psychosocial or perceived stress^29–31^. There are several major findings in this report. Firstly, this study demonstrated that autophagy was substantially enhanced in the intestinal tissue of CRH-administrated IBD mice, suggesting a negative role of autophagy in psychosocial stress-related IBD. Furthermore, we confirmed this phenotype by showing that autophagic blocker successfully abolished the action of CRH in IBD model and in LPS-treated BMDM. Second, CRH administration markedly changed the gut microbiota in the intestine of IBD mice. Finally, our results clearly indicated that the psychosocial stress was positively associated with autophagy marker in intestinal samples from IBD patients. All of these data imply that autophagy plays a crucial role in the pathophysiology of psychosocial stress-related IBD by regulating the interaction between intestinal mucosal barrier and microbiota and promoting macrophage M1/M2 polarization (Supplemental Fig. 3).

CRH was highly produced in the mucosal epithelial cells of colonic tissues from IBD patients both in protein and mRNA levels and was involved in the damage to mucosal barrier function and induction of colonic hyper-permeability, resulting in the stress-related intestinal disorders^32^. The present study used CRH to induce stress and showed that peripheral administration of CRH in IBD mice significantly deteriorated the severity of IBD in the extent of weight loss, bloody diarrhea, shortened colon length, and colonic inflammatory reaction. Our results showed that there was a moderate correlation between the perceived stress and IBD severity in patients. In addition, this study for the first time reported the influence of peripheral CRH in the homeostasis of gut microbiota. Since several CRHR1 antagonists, such as non-peptide CRHR1 antagonists, have been proven to be effective for the alleviation of IBD via the blockade of colonic hypersensitivity induced by colonic inflammatory reaction, thus providing a novel therapeutic options in the treatment of stress-aggravated IBD^33,34^, whether these antagonisms affect autophagy and gut microbiota may be interesting questions. However, it should be noted that unlike CRHR1, the inhibition of CRHR2 might led to the reverse effects, indicating the possible problems in the development of therapies targeting on peripheral CRH for the treatment of IBD^35^.

Autophagy is regarded as a vital cellular catabolic pathway. In IBD, previous studies reported that knocking out autophagy-related key genes, including Atg4B/autophagin-1, Atg16L1, and activating transcription factor 4 (ATF4) significantly deteriorated the severity of IBD in animal models, suggesting that baseline autophagy was vital for the maintenance of intestinal homeostasis and the function of the intestinal defensive barrier^36–39^. As a basic leucine zipper transcription factor, activating transcription factor 4 (ATF4) could regulate inflammatory response^40^, amino acid metabolism^41,42^, autophagy^43^, and endoplasmic reticulum stress^44^, which is expressed in many tissues, including the intestine. Hu et al.^45^ revealed that ATF4 was significantly decreased in the inflamed intestinal mucosa of patients with active IBD, while they also illustrated that disruption of ATF4 most likely was linked to the ileal form of CD but not the colonic tissue. In our study, we mainly examined the level of ATF4 in the colonic tissues but no significant differences were found among the control, the mild and moderate IBD and the severe IBD groups (Fig. 1f), which was favored by the research conducted by Bretin et al.^43^. Similarly, no changes were found in DSS-induced mice (Supplementary Fig. 1). Taken together, we proposed that ATF4 might not take part in the deterioration of the severity of IBD induced by CRH.

In addition, autophagy has been demonstrated to play a positive role in the alleviation of IBD^46,47^.However, it was also demonstrated that over-activation of autophagy might aggravate IBD through the induction of autophagic cell death, thus leading to the disruption of the intestinal barrier and the excessive production of proinflammatory cytokines^48–51^. Chloroquine, a classic autophagy inhibitor, has long been proven to act as a potential therapy for the treatment of IBD via the regulation of cellular metabolism and modulation of inflammatory reaction^48,52–54^. Consistently, in the current study, we observed an increase in autophagy in colonic tissues during the occurrence of IBD and peripheral administration of CRH further enhanced the level of autophagy accompanied with the aggravation of IBD severity. The similar trend was also validated by our human sample. Furthermore, the administration of chloroquine for the blockade of over-induced autophagy process largely attenuated the detrimental effects of aggravated the severity and colonic inflammatory infiltration of IBD led to by peripheral administration of CRH. Those data indicated the therapeutic effect of blockade of autophagy in IBD caused by peripheral CRH. Paneth cell, a member of IEC, is an important component of intestinal defensive system through the secretion of various kinds of antimicrobial peptides, including HD-5, HD-6, and lysozyme, which largely influence the intestinal inflammatory and immune responses^55,56^. This study demonstrated that the occurrence of IBD led to the increase in Paneth cell metaplasia in the left colon. The challenge of stress induced by peripheral administration of CRH further led to the increase in Paneth cell metaplasia, contributing to the severity of IBD. In addition, colonic autophagy was demonstrated to be involved in DSS-induced Paneth cell metaplasia according to our findings. The application of chloroquine for the blockade of the autophagy process largely attenuated Paneth cell metaplasia, while the administration of rapamycin for the induction of autophagy led to the reverse effects.

The microenvironment of the gut forms a proper microbiota habitat that has been demonstrated to influence the occurrence of many kinds of digestive diseases^57,58^. The disturbance of intestinal microbiota homeostasis has been widely considered to be closely associated with the pathogenesis and progression of IBD^59^. This present study demonstrated that peripheral administration of CRH led to further disturbance of gut microbiota homeostasis, indicating that restoring microbiota homeostasis was vital for the treatment of stress-aggravated IBD. In addition, the polarization of the M1 proinflammatory phenotype has been considered as an important factor for the onset and development of IBD, thus reducing the ratio of M1/M2 was effective for the alleviation of over-whelming inflammatory reaction^60,61^. This current study demonstrated that peripheral administration of CRH led to the polarization of the M1 phenotype in mice and that the patients with higher CPSS also presented the similar augmentation of the M1 phenotype. The CRH-induced M1 polarization process was involved in autophagy, through our findings that blocking autophagy by chloroquine significantly attenuated the effect.

Taken together, we demonstrated that stress factor aggravated IBD via activating autophagy. Our findings might lead to a better understanding of the pathogenesis and progression of IBD and provide a novel insight on the autophagy in treatment of IBD.

## Materials and methods

### Collection and analysis of human IBD samples

The study regarding the role of stress on severity and autophagy of IBD was conducted at the Digestive Endoscopy Center of Changhai Hospital, an open-access academic tertiary endoscopy center. According to the improved Mayo score system^62^, colonic tissue biopsy was performed during colonoscopy in healthy control and patients with severe and mild/moderate IBD between February 2018 and May 2018. The colonoscopy was performed as described in our previous study^63^. During the procedure, we collected the basic demographic characteristics of IBD patients and assessed the level of perceived stress using the CPSS. The CPSS is a ten-item instrument designed to measure the degree to which the subject has perceived life as unpredictable, uncontrollable, and overloading during the previous month^64^. Apart from these parameters, the endoscopic score was evaluated using the endoscopy ranging from 0 to 4, which corresponded to the improved Mayo score system. Subsequently, the tissue was fixed in 4% (w/v) paraformaldehyde overnight and embedded in paraffin. The severity of inflammation was assessed via hematoxylin-eosin staining, while the level of autophagy and the number of M1 macrophage were estimated by immunohistochemical staining. The levels of autophagy-related proteins were further analyzed via western blotting. The protocol of this study was carried out according to the principles of the Declaration of Helsinki and approved by the Medical Ethics Committee in Shanghai Changhai Hospital, Shanghai, China. Written informed consent was obtained from all of the participants before enrollment.

### Animal care and use

C57BL/6 mice were purchased from Shanghai Super-B&K Laboratory Animal Corp., Ltd. (Shanghai, China). Mice were kept at 22 °C under a 12-h light/dark cycle with unlimited access to water and a standard rodent diet. During the experiments, the mice were anesthetized with phenobarbital sodium (60 mg/kg, i.p.) and were euthanized using cervical dislocation under anesthesia unless indicated otherwise. All of the experiments were approved and conducted in accordance with the guidelines of the Animal Care Committee of Second Military Medical University/ Naval Medical University, Shanghai, China.

### Induction of IBD in mice

The IBD model was induced in C57BL/6 mice with 3% DSS (mol. wt. 36,000 to 50,000 kDa, MP Biomedicals LLC, Santa Ana, CA, USA) dissolved in drinking water given ad libitum (days 1–6) as previously described^65^. For some groups, CRH (50 μg/kg body weight, Tocris, Ellisville, MO, USA), chloroquine (60 mg/kg body weight, Sigma-Aldrich, St. Louis, MO, USA), and/or rapamycin (1.25 mg/kg body weight, Selleck Chemicals, Houston, TX, USA) were intraperitoneally injected from day 1 to day 6. Saline was injected as vehicle.

### Clinical score and histological analysis in mice

Body weight, the presence of occult or gross blood per rectum, stool consistency, and colon length were determined by two investigators blinded to the treatment groups. A scoring system was used to assess diarrhea and the presence of occult or overt blood in the stool^66^. Body weight changes were shown as loss of baseline body weight. Postmortem, the colon was removed, and pieces of colonic tissue were used for ex vivo analysis. For histology, rings of certain parts of the colon were fixed in 4% buffered formalin and embedded in paraffin. Sections were stained with H&E according to standard protocols. Histological scoring was performed in a blinded way by a pathologist. Focally increased the number of inflammatory cells in the lamina propria was scored as 1, the confluence of inflammatory cells extending into the submucosa as 2, and the transmural extension of the infiltrate as 3. For tissue damage, discrete lymphoepithelial lesions were scored as 1, mucosal erosions as 2, and extensive mucosal damage and/or extension through the deeper structures of the bowel wall as 3. The two equally weighted subscores (cell infiltration and tissue damage) were added and the combined histological colitis severity score ranged from 0 to 6.

### Cell culture and treatment

Murine BMDM were obtained through the incubation of bone marrow cells as previously described^67^. In brief, bone marrow was flushed out from femurs and tibias, cultured, and differentiated in bone marrow growth medium comprised of Dulbecco’s modified Eagle’s medium (Gibco, Grand Island, NY, USA) supplemented with 10% fetal bovine serum (Gibco, Grand Island, NY, USA), 30% L929 cell-conditioned media (source of macrophage-colony stimulating factor, M-CSF), and penicillin/streptomycin at 37 °C in a humidified incubator with 5% CO_2_. The bone marrow growth medium was renewed every 2 days. After 7-day cultivation, fresh medium was replaced. For certain groups, lipopolysaccharide (LPS) (100 ng/ml, Sigma-Aldrich, St. Louis, MO, USA), CRH (10^−8 ^M, Tocris, Ellisville, MO, USA) and/or chloroquine (10 μM, Sigma-Aldrich, St. Louis, MO) were administrated for the stimulation of 12 h.

### Facial action coding system (FACS) analysis

After the aforementioned treatment, murine BMDM were digested and washed with PBS three times. Cells were collected and incubated with CD68 antibody (1:500, eBioscience, San Diego, CA, USA) or CD206 antibody (1:500, eBioscience, San Diego, CA, USA) for 30 min at room temperature. Mouse IgG1 kappa was used as negative control (eBioscience, San Diego, CA, USA). The ratio of CD68^+^ cells and CD206^+^ cells were analyzed using a flow cytometer (Thermo Fisher Scientific, Waltham, MA, USA).

### Western blotting

Proteins were extracted from the colonic tissues or murine BMDM using a standard extraction reagent supplemented with the protease inhibitor (KANGCHEN; Shanghai, China). The protein concentration was determined using a bicinchoninic acid protein assay kit (Beyotime Institute of Biotechnology, Haimen, China). Proteins were then separated using sodium dodecyl sulfate polyacrylamide gel electrophoresis and electro-transferred to nitrocellulose membranes as described previously^68^, and incubated with a primary antibody overnight at 4 °C. The samples were then incubated with an IRDye800CW-conjugated secondary antibody (Rockland, Gilbertsville, PA, USA) for 1 h at 25 °C. The image was acquired with the Odyssey infrared imaging system (Li-Cor Biosciences, Lincoln, NE, USA). All of the immunoblotting experiments were repeated at least five times. The following primary antibodies were used: Beclin-1 antibody (1:500; Cell Signaling Technology, Danvers, MA, USA), light chain 3 (LC3) antibody (1:500; Novus Biologicals, Littleton, CO, USA), and p62 antibody (1:500; Cell Signaling Technology, Danvers, MA, USA), CD68 antibody (1:300; Proteintech Group Inc., Chicago, IL, USA), and CD206 antibody (1:500; Proteintech Group Inc., Chicago, IL, USA).

### Real-time polymerase chain reaction (real-time PCR)

Total RNA from colon tissues and murine BMDM was isolated by TRIzol (Invitrogen, Carlsbad, CA, USA). The first-strand cDNA was synthesized using PrimeScript RT Master Mix (Takara, Otsu, Shiga, Japan) and a 2^ΔΔCT^ method was used to analyze the results of PCR with GAPDH as the internal reference. 7500 real-time PCR System and the Fast Start Universal SYBR Green Master (Roche, Basel, Switzerland) were used for real-time PCR. Primers used were listed as follows: CD68: forward, 5′-CCTCTTGCTGCCTCTCATCATTGG-3′ and reverse, 5′-GGCTGGTAGGTTGATTGTCGTCTG-3′; CD206: forward, 5′-ACCTGGCAAGTATCCACAGCATTG-3′ and reverse, 5′-TGTTGTTCTCATGGCTTGGCTCTC-3′; GAPDH: forward, 5′-GTATGACTCCACTCACGGCAAA-3′ and reverse, 5′-GGTCTCGCTCCTGGAAGATG-3′.

### Immunofluorescence staining

Colonic tissues were fixed in 4% (w/v) paraformaldehyde overnight and embedded in paraffin. Five μm sections were then sent for dewaxing and rehydration. After the sections were blocked with 5% bovine serum albumin in PBS for 2 h, they were incubated with lysozyme antibody (1:250, Abcam, Cambridge, MA, USA), CD68 antibody (1:100, Proteintech Group Inc., Chicago, IL, USA), CD206 antibody (1:100,; Proteintech Group Inc., Chicago, IL, USA) or LC3 antibody (1:200, Novus Biologicals, Littleton, CO, USA) overnight at 4 °C. After washing three times with PBS, the sections were stained by Alexa-488- or Alexa-Cy3-labeled secondary antibody (1:500, Jackson ImmunoResearch Inc., West Grove, PA, USA) for 30 min at 37 °C. After washing, the slides were mounted with Vectashield mounting medium containing 4′,6-Diamidino-2-Phenylindole (DAPI; Vector Laboratories, Burlingame, CA, USA) and colocalization was observed using a confocal laser scanning microscope (Fluoview FV1000, Olympus, Tokyo, Japan). In this study, the experiments were performed in a double-blind manner.

### Immunohistochemical staining

Colonic tissues were fixed in 4% (w/v) paraformaldehyde overnight and embedded in paraffin. Five micrometer sections were then sent for dewaxing and rehydration. The sections were blocked by goat serum and incubated in lysozyme antibody (1:2000, Abcam, Cambridge, MA, USA), LC3 antibody (1:200, Servicebio technology, Wuhan, China), and CD68 antibody (1:100, Servicebio technology, Wuhan, China). After washing three times with PBS, the sections were incubated with horseradish peroxidase-conjugated secondary antibodies. Staining is visualized using substrate DAB. Images were obtained using a digital microscope (Leica, TCS SP8, Leica, Biberach, Germany).

### Enzyme-linked immunosorbent assay (ELISA)

The levels of tumor necrosis factor-α (TNF-α), interleukin (IL)-18, IL-7, myeloperoxidase (MPO), and MPO activity in the colon tissues were quantified using commercial ELISA kits according to the manuscript’s instruction (R&D system, Minneapolis, MN, USA).

### Assessment of MPO activity

O-dianisidine method was used to assess MPO activity, which represented the neutrophil infiltration of inflammatory colonic mucous. Proteins were extracted from the colonic tissues and were used to assess the level of MPO according to the manufacturer’s instructions (Jiancheng, Nanjing, China). The results were shown as activity units per milligram tissue.

### Transmission electron microscopy

Colonic tissues were separated and fixed overnight at 4 °C in 2.5% glutaraldehyde in 0.1 M PBS, and then post-fixed in 1% buffered osmium tetroxide for 2 h. Specimens were processed via a routine procedure and examined under a transmission electron microscope (H-700; Hitachi, Tokyo, Japan).

### Gut microbiota determination

Gut microbiota was determined using 16S ribosomal RNA gene sequencing technology. For sample collection and DNA extraction, fecal samples were collected from experimental mice and frozen at −80 °C within 3 h after sampling. DNA extraction was performed using a QIAamp Fast DNA Stool Mini Kit (Qiagen, Hilden, Germany). The concentration of bacterial DNA was measured using NanoDrop 2000 (Thermo Scientific, Pittsburgh, PA, USA). Then, the 16S ribosomal RNA gene sequencing was applied to detect the bacterial DNA. The V3–V4 region of the bacteria’s 16S ribosomal RNA (rRNA) gene was amplified by PCR with barcode-indexed primers (338F and 806R), using FastPfu Polymerase. Amplicons were then purified by gel extraction (AxyPrep DNA GelExtraction Kit, Axygen Biosciences, Union City, CA, USA) and were quantified using QuantiFluor-ST (Promega, Madison, WI, USA). The purified amplicons were pooled in equimolar concentrations, and paired-end sequencing was performed using an Illumina MiSeq instrument (Illumina, San Diego, CA, USA).

### Microbial and imputed metagenomic analysis

The 16S rRNA sequencing data were processed by the Quantitative Insights Into Microbial Ecology platform (V.1.9.1) and MegaBLAST search was conducted to align the reads of the Operational taxonomic units against the reference sequences in the National Center for Biotechnology Information 16S rRNA database as described previously^69^. For metagenomic analysis, the metagenomes of gut microbiome were imputed from 16S rRNA sequences with PICRUSt (Phylogenetic Investigation of Communities by Reconstruction of Unobserved States). The gene contents were predicted for each sample and pathways present in less than 10% of the samples were not included in the comparison analysis.

### Statistical analysis

Data were presented as means ± SEM. A two-way analysis of variance (ANOVA) followed by Bonferroni’s post-hoc test for repeated measures were used for the analysis of the statistical significance of the colitis clinical scores between treatments. For other analysis, a Kruskal–Wallis test followed by Dunn’s post-hoc test and one-way ANOVA followed by Bonferroni’s post-hoc test was used to determine nonparametric data and continuous variables, respectively. A *P*-value < 0.05 was considered statistically significant. Data were analyzed with SPSS 21.0 K for Windows (SPSS, Chicago, IL, USA).

## Supplementary information

Supplementary materials
