# Supplementary material for: Intestinal autophagy links psychosocial stress with gut microbiota to promote inflammatory bowel disease
Source: Cell Death Dis. 2019 Sep 30;10(6):391. doi: 10.1038/s41419-019-1634-x (PMC6766473; doi:10.1038/s41419-019-1634-x)
Supplement: Supplementary file 1 — Supplementary materials [file 41419_2019_1634_MOESM1_ESM.docx]

**Supplemental Figure 1**


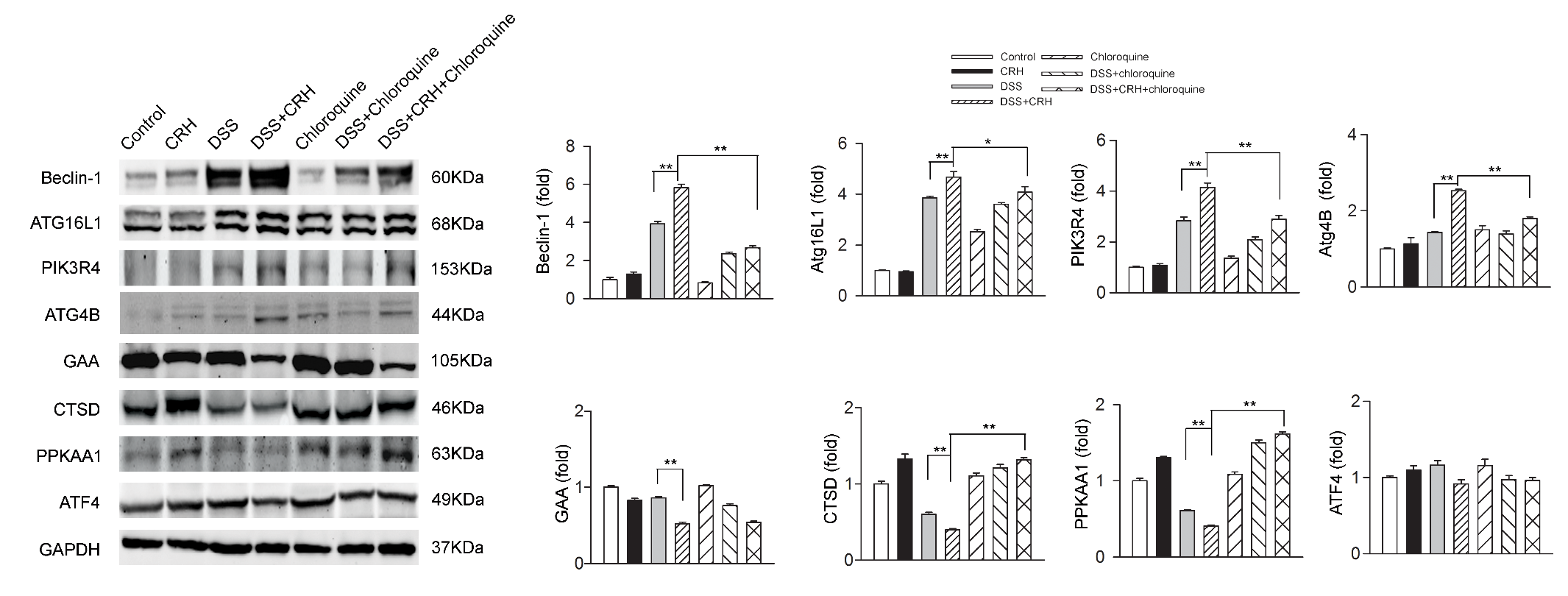


**Supplemental Figure 1. Chloroquine attenuates CRH-induced enhancement of autophagy in the left colon from IBD mice.**

DSS (3%) was given to C57BL/6 mice for 7 days while water was given to the control group. CRH (10 mg/kg body weight) and/or chloroquine (60 mg/kg body weight) was intraperitoneally given and saline was injected as vehicle. The left edge of the left colon was separated and Western blotting was conducted for the analysis of beclin-1, Atg16L1, PIK3R4, Atg4B, GAA, CTSD, PPKAA1 and ATF4. Compared to the DSS+Vehicle group, the DSS+CRH group had increased levels of Beclin-1, Atg16L1, PIK3R4 and Atg4B but decreased levels of GAA, CTSD and PPKAA1, while the administration of chloroquine largely attenuated the effects of CRH in beclin-1, Atg16L1, PIK3R4, Atg4B, CTSD and PPKAA1. The level of ATF4 was not significantly changed (n=6 per group). ^*^P<0.05, ^**^P<0.01.

**Supplemental Figure 2**


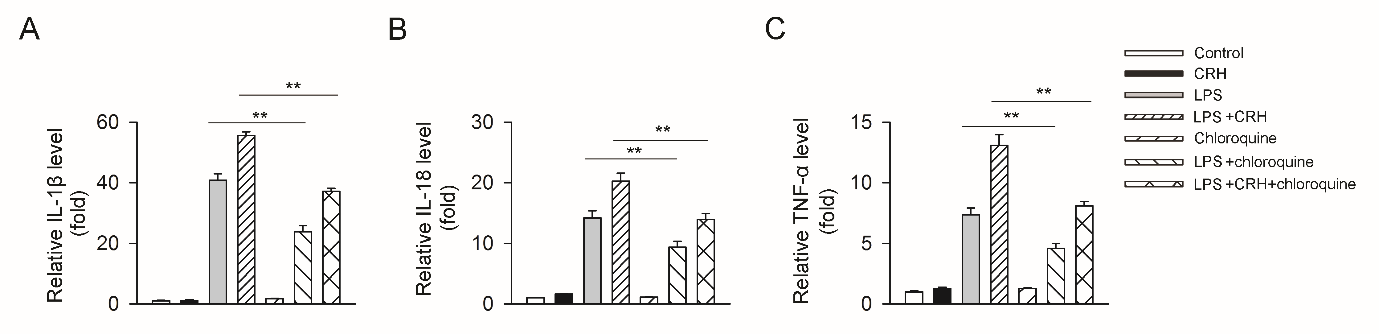


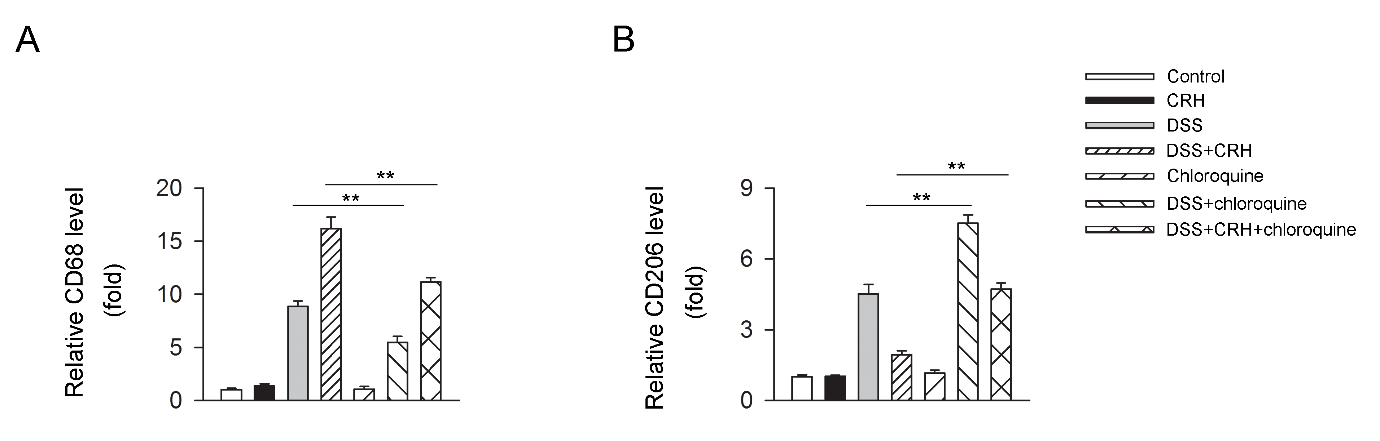


**Supplemental Figure 2. Chloroquine attenuates CRH-induced enhancement of autophagy in murine BMDMs under the challenge of LPS.**

Murine BMDMs were obtained and stimulated with LPS, CRH and/or chloroquine for 12 h. Realtime PCR was used for the detection of the levels of CD68 (A) and CD206 (B) in murine BMDMs. Compared to the LPS+Vehicle group, the LPS+CRH group had increased levels of CD68 and decreased levels of CD206, while the administration of chloroquine largely attenuated the effect of CRH (n=6 per group). ^**^P<0.01.

**Supplemental Figure 3**


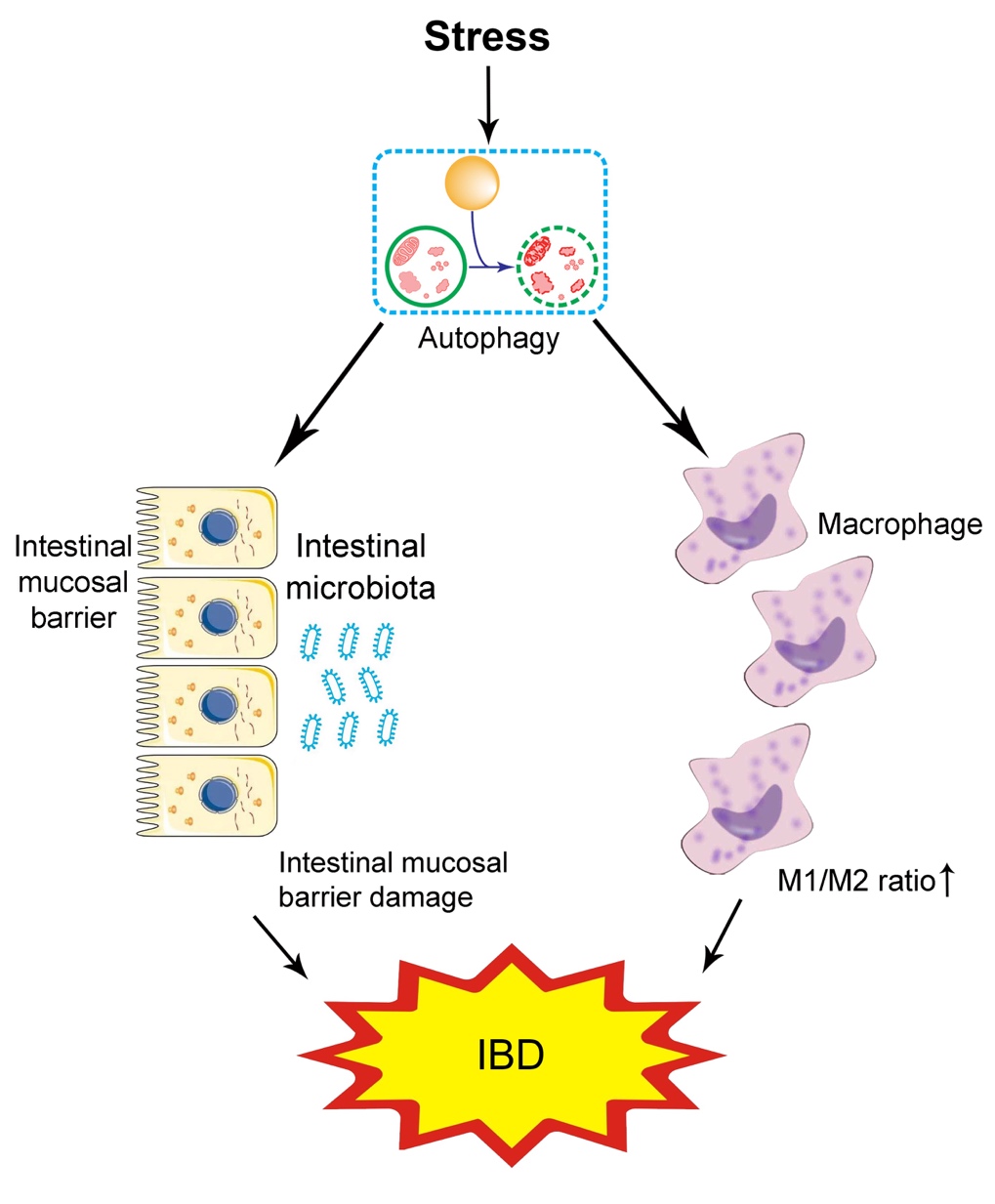


**Supplemental Figure 3.** A proposed working model for the role of autophagy in psychosocial stress-related IBD.
